# Molecular Dynamics Study on Structure, Vibrational Properties, and Transport Coefficients of Liquid Alumina

**DOI:** 10.3390/ma15238370

**Published:** 2022-11-24

**Authors:** Xiaolin Zhou, Yufeng Zhou, Ya Deng, Yumin Zhang

**Affiliations:** 1 National Key Laboratory of Science and Technology on Advanced Composites in Special Environments, Harbin Institute of Technology, Harbin 150080, China; 2 Center for Composite Materials and Structures, Harbin Institute of Technology, Harbin 150080, China

**Keywords:** liquid alumina, structural property, vibrational density of states, self-diffusion coefficient, thermal conductivity, viscosity

## Abstract

The structure, vibrational density of states, and transport coefficients of liquid alumina were studied using molecular dynamics simulations. At the temperature of 2500 K, 3000 K, 3500 K, and 4000 K, systems with three different densities were constructed, respectively, including the configurations with densities of 2.81 g/cm^3^ and 3.17 g/cm^3^, and the relaxed ones with nearly zero pressure at each temperature. With the changes in temperature or density, the transformations on the structural, vibrational and transport properties were discussed. The Born–Mayer–Huggins type of atomic interactions was used, with newly optimized parameters. The analysis of the interatomic correlations indicated that the short-range order of liquid alumina was mainly constructed by AlO_4_ tetrahedra, also a certain number of AlO_3_ and AlO_5_ was present. Meanwhile, the structural transitions on the elemental units occurred as either the temperature or density increased. Two primary frequency bands were observed in each vibrational density of states spectrum, with the higher frequency bands produced by the O atom vibrations, and the lower frequency ones generated by the Al atom vibrations. Self-diffusion coefficients were estimated using the linear behavior of the mean-squared displacement at long time, while by using the Green–Kubo relation during equilibrium molecular dynamics simulations, thermal conductivities and viscosities were calculated. Significantly, the viscosity at 2500 K with a density of 2.81 g/cm^3^ was equal to 25.23 mPa s, which was very close to the experimental finding.

## 1. Introduction

Alumina is a significant ceramic material with various technical applications, such as high-temperature resistant ceramics [1,2,3], catalysts [4,5,6], abrasives [7,8], coatings [9,10,11], insulators [12,13,14] and capacitors [15,16]. Meanwhile, molten alumina has received much attention, due to its utilization in analyzing the performance of aluminum-fueled rocket engine effluent [17], and also in the production of large sapphire single crystals [18,19].

Understandings of the origins of the outstanding macroscopic features can be promoted by the knowledge of the microscopic structures. The high melting point of alumina (up to 2327 K) [20], however, frequently caused contamination from the container throughout the experiment, reducing the accuracy of the obtained structural information. With the use of X-ray diffraction [21,22,23], neutron scattering measurements [24,25], nuclear magnetic resonance measurements (NMR) [26,27,28], and aerodynamic-levitation and laser-heating techniques [29], some processes in the structural investigations of molten alumina have been achieved. Most of the experiments obtained the coordination numbers of Al-O pairs between 4 and 5. However, there were also some disagreements; for example, the measurement conducted by Waseda et al. [22] using X-ray diffraction at 2363 K concluded that the coordination number *Z*_Al-O_ was equal to 5.6, indicating that the elemental units for the construction of liquid alumina were octahedral networks. Besides the structural information, the physical properties of molten alumina, for example, electrical conductivity [30], heat capacity [31], surface tension [32,33], emissivity [34], and viscosity [32], were investigated by non-contract measurements.

However, the experimental data were usually inadequate and difficult to reproduce because of the challenges experienced during the experiment processes. An alternative method was to estimate the characteristics of liquid alumina using first-principle calculations or molecular dynamics (MD) simulations. While it was discovered that liquid alumina was mostly made up of AlO_4_ tetrahedrons [29,35,36,37,38,39], others suggested that AlO_5_ may have also been present in significant amounts [40,41,42]. Even Hemmati [43] claimed that liquid alumina was mostly composed of AlO_5_ units. It was also evident from the MD simulations that the pore aggregation underwent a continuous transition as the pressure increased [41,44,45,46]. Additionally, by using the slopes of the mean-squared displacements, the self-diffusion coefficients were determined [36,37,40,41]. The specific heat, vibrational density of states, and ionic conductivity were examined at the temperature of 2600 K basing on an atomic interaction consisting of two- and three-body terms by Vashishta et al. [35]. Meanwhile, the charge-transport properties of liquid alumina were discussed in detail by Gheribi et al. [47].

It is worth noting that most computational research on liquid alumina was performed at high or ambient pressures, and the relaxation configurations at particularly zero pressure have been less well understood. Furthermore, the earlier studies on liquid alumina have primarily aimed at the structural characteristics, with only a small number of them being concerned with the physical properties, particularly given the fact that a detailed exploration of transport properties has rarely been reported.

Therefore, in this study, the configurations with nearly zero or a certain pressure were all investigated. Furthermore, besides the structure and vibrational density of states, the transport coefficients of liquid alumina were carefully examined in this research. Using the newly optimized parameters [48], the examinations were conducted by classical MD simulations based on Born–Mayer–Huggins (BMH) atomic interactions. Systems with three different densities were established at temperatures of 2500 K, 3000 K, 3500 K, and 4000 K, respectively. At each temperature, the examinations were performed on not only the configurations with densities of 2.81 g/cm^3^ and 3.17 g/cm^3^ (with some pressures), but also the relaxed ones with nearly zero pressure. Moreover, transformations in structural, vibrational, and transport characteristics were discussed in relation to the changes in both density and temperature.

## 2. Simulation Methods

**Figure 1:**
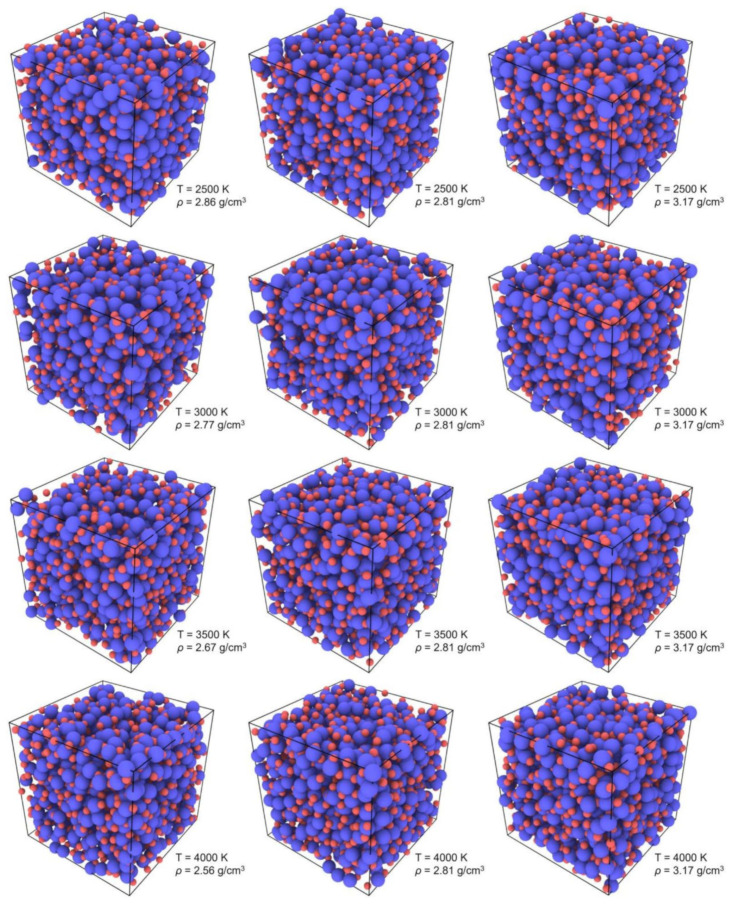
Snapshots of the considered configurations in this study at the temperatures of 2500 K, 3000 K, 3500 K, and 4000 K, with three different densities at each temperature (the blue particles: Al atoms, the red particles: O atoms).

2000 atoms (1200 O and 800 Al) were packed randomly into a basic cube, creating a system with periodic boundary conditions in x, y and z directions. Using the Verlet algorithm in the velocity form with a timestep of 1.0 × 10^−3^ ps, Newton’s equations of motion were integrated. The short-range interaction was cut off at 8.0 Å, and the Coulomb interaction was calculated by the Ewald summation method. After being equilibrated in the isobaric–isothermal ensemble (NPT) at zero pressure and the temperature of 4500 K, the initial configuration was cooled down to 2500 K with a cooling rate of 1 K/ps. The corresponding configuration was taken at intervals of 500 K, and it was equilibrated for 1000 ps at a constant temperature and zero pressure. Since the object of this study was liquid alumina, the simulation conducted temperature was required to be higher than the melting point of 2327 K [20]. Therefore, the simulations were carried out at 2500 K, 3000 K, 3500 K, and 4000 K, respectively. Furthermore, in order to compare the structure, vibrational density of states and transport coefficients of liquid alumina as a function of density, two configurations with densities of 2.81 g/cm^3^ and 3.17 g/cm^3^ were produced by adjusting the volume of the simulation box at each temperature. In particular, the density of 2.81 g/cm^3^ was experimentally recorded from neutron scattering measurements [25], while many previous simulations of liquid alumina were also performed on the density of 3.17 g/cm^3^, which could be utilized to check the reliability of the simulation results in this paper by the comparisons with the reported results, including those obtained from both experiments and simulations. Moreover, at each temperature, configurations with densities of 2.81 g/cm^3^ and 3.17 g/cm^3^ as well as those with anticipated densities at nearly zero pressure were investigated. The snapshots of the considered configurations at different densities and temperatures are shown in Figure 1. Classical MD simulations were carried out with the LAMMPS code [49] (version: stable_3Mar2020) by applying the Born–Mayer–Huggins (BMH) potential, and atomic interactions between O and Al particles were provided by

(1) $$V\left(r_{\alpha \beta }\right)=\frac{q_{\alpha }q_{\beta }}{r_{\alpha \beta }}+A_{\alpha \beta }\exp\left(\frac{\sigma_{\alpha \beta }-r_{\alpha \beta }}{\rho_{\alpha \beta }}\right)-{\left(\frac{C_{\alpha \beta }}{r_{\alpha \beta }}\right)}^{6}-{\left(\frac{D_{\alpha \beta }}{r_{\alpha \beta }}\right)}^{8}$$

with *α* or *β* = O or Al, and *r_αβ_* being the distance between the centers of the particle *α* and the particle *β*. *q_α_* and *q_β_* are the effective charges in the first term of the pair potential, which is associated with the long-range Coulomb. The Born repulsive is shown in the second term, while the dipolar expansion corresponds to the third and the fourth ones, though just the van der Waals term was calculated. Recently, Bouhadja et al. [48] adjusted the parameters of *A_αβ_*, *σ_αβ_*, *ρ_αβ,_* and *C_αβ_* in Equation (1), as displayed in Table 1.

The atomic correlations were used to determine the short-range order of the structural characteristics of liquid alumina, including the pair distribution function (*g*(*r*)) and the coordination number as well as the bond-angle distribution. These methods were commonly employed for the analysis of microscopic structures and have been proven to be efficient [50,51,52,53,54]. The partial pair distribution function (*g_αβ_*(*r*), PPDF) is shown as Equation (2):

(2) $$g_{\alpha \beta }\left(r\right)=\frac{N_{\beta }}{V}\cdot \frac{\left\langle n_{\alpha \beta }\left(r,\;r+\Delta r\right)\right\rangle }{4\pi r^{2}\Delta r}\left(i,\;j=\mathrm{Al}\;\mathrm{or}\;O\right)$$

where *N_β_* is the number of the particle *β*, *V* represents the volume of the system, $n_{\alpha \beta }\left(r,\;r+\Delta r\right)\;$ represents the average number of the *β* particles spanning the space from *r* to *r+*Δ*r*. The total pair distribution function (PDF) was calculated as Equation (3):

(3) $$g\left(r\right)=\underset{\alpha ,\beta }{\sum }\frac{N_{\alpha }}{N}\cdot \frac{N_{\beta }}{N}\cdot g_{\alpha \beta }\left(r\right)$$

By using the minimum following the first peak of *g_αβ_*(*r*) as a cutoff radius (*R*), which was taken as *R*_Al-O_ = 2.38 Å, *R*_Al-Al_ = 3.76 Å and *R*_O-O_ = 3.65 Å, the coordination numbers *Z_αβ_*(*R*), characterizing the mean number of *β* particles surrounding an *α* particle, were calculated as Equation (4):

(4) $$Z_{\alpha \beta }\left(R\right)=\frac{4\pi N_{\beta }}{V}\int_{0}^{R}g_{\alpha \beta }\left(r\right)r^{2}dr$$

Regarding the dynamics, the vibrational density of states (VDOS) and transport coefficients (self-diffusion coefficient, thermal conductivity and viscosity) were estimated. For each configuration, five independent simulations with various beginning momenta were performed to decrease the statistical errors, since the transport coefficients are sensitive to the initial conditions. Additionally, the results were calculated by averaging the standard deviations over the individual simulations.

By conducting a Fourier transform on the velocity autocorrelation function (*Z*(*t*)), VDOS was determined as Equation (5):

(5) $$G\left(\omega \right)=\frac{\int_{0}^{\infty }Z\left(t\right)e^{-i\omega t}dt}{2\pi }$$

The self-diffusion coefficient (*D*), in this study, was computed from the linear behavior of the mean-squared displacement (MSD) over a long period, as displayed in Equation (6). This method has been widely used in the calculations of self-diffusion coefficients for many different systems, such as drugs [50], gases [55,56] nanoparticles [57,58], and liquids [59,60]. Moreover, the obtained results often showed to be stable due to the long correlation time.

(6) $$D_{i}=\frac{\underset{t\to \infty }{\lim}\frac{d}{dt}\langle \sum_{i=1}^{N}{\left|r_{i}\left(t\right)-r_{i}\left(0\right)\right|}^{2}\rangle }{6N}$$

where *r_α_*(*t*) denotes the vector of the position related to particle *α* when at time *t*, and *N* denotes the number of the particle *α*.

Since liquid alumina was a homogeneous system, thermal conductivity (*κ*) and viscosity (*η*) could be estimated by the equilibrium molecular dynamics (EMD) simulations with the Green–Kubo relation. The integral of time of the heat-current autocorrelation function (HCACF) was used to calculate the thermal conductivity, while that of the stress autocorrelation function (SACF) was used to evaluate the viscosity. The definition of the heat-current (*J*) is:

(7) $$J=\frac{1}{V}\left(\underset{\alpha }{\sum }e_{\alpha }v_{\alpha }-\underset{\alpha }{\sum }S_{\alpha }v_{\alpha }\right)$$

where $v_{\alpha }$ denotes the velocity of the particle *α*, *e_α_* represents the potential and kinetic energy of the particle *α*, and *S_α_* denotes the atomic stress of the particle *α*. Subsequently, using the Green–Kubo relation, EMD simulations were carried out to compute *κ*:

(8) $$\kappa \left(t\right)=\frac{V\cdot \int_{0}^{t}\langle J\left(t\right)\cdot J\left(0\right)\rangle dt}{3k_{B}T^{2}}$$

where *k_B_* represents the Boltzmann constant, and *T* means the equilibrium temperature.

The component of molecular stress tensor *P_ij_* for SACF (*ij* = *xy*, *xz*, and *yz* directions) is defined as:

(9) $$P_{ij}=\frac{\left[m_{\alpha }\cdot v_{\alpha i}\cdot v_{\alpha j}+\sum_{\beta \neq \alpha }r_{\alpha \beta }\cdot F_{\alpha \beta }\right]}{V}$$

where *m_α_* is the mass of the particle *α*, *v_αi_* and *v_αj_* are the velocities of the particle *α*, *r_αβ_* denotes the relative position of the particle *α* about particle *β*, and *F_αβ_* represents the force operating on the particle *α* as a result of the interaction with particle *β*. Then *η* was computed by EMD simulation as Equation (10):

(10) $$\eta \left(t\right)=\frac{V\cdot \int_{0}^{t}\langle P_{ij}\left(t\right)\cdot P_{ij}\left(0\right)\rangle dt}{k_{B}T}$$

## 3. Results and Discussion

In this study, the calculated zero-pressure densities were equal to 2.86 g/cm^3^, 2.77 g/cm^3^, 2.67 g/cm^3^, and 2.56 g/cm^3^, respectively, at 2500 K, 3000 K, 3500 K, and 4000 K, decreasing with the increasing temperature. Notably, the predicted density values at 2500 K and 3000 K were consistent with the experimental values of 2.81 g/cm^3^ and 2.78 g/cm^3^, which were measured, respectively, by the neutron scattering measurements [25] and aerodynamic-levitation technique [29], indicating the efficiency of the optimized parameters of the BMH potential.

### 3.1. Structural Properties

**Figure 2:**
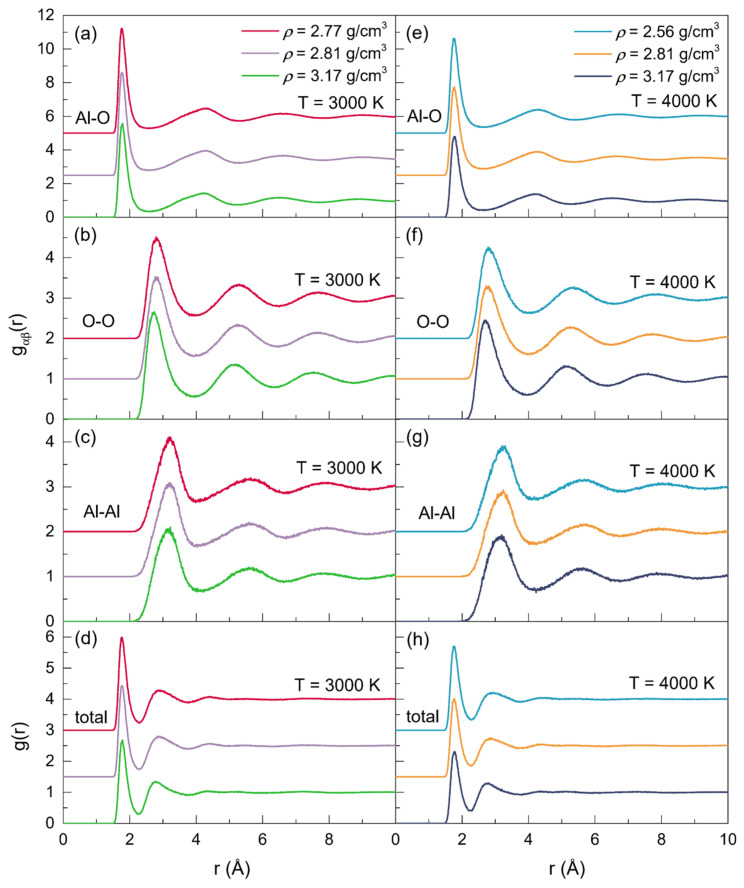
Partial and total pair distribution functions for the configurations of liquid alumina with three densities, respectively, at 3000 K and 4000 K.

At 2500 K, 3000 K, 3500 K, and 4000 K, the position (*r_αβ_*) and the full width at half maximum (FWHM) of the initial peak in each PPDF for the configurations with three different densities are shown in Table 2. The positions of the first peaks associated with the Al-O, Al-Al, and O-O correlations all agreed well with the experimental, ab initio and MD simulation results. As displayed in Figure 2, when at a fixed temperature, the initial peaks in the *g*_Al-O_ correlations (Figure 2a,e) shifted to larger values with the density increasing, while those in the *g*_O-O_ (Figure 2b,f) and *g*_Al-Al_ (Figure 2c,g) correlations moved toward smaller values, demonstrating the increase in *r*_Al-O_, and the decrease in *r*_O-O_ and *r*_Al-Al_. Meanwhile, the initial peaks in *g*_Al-Al_ and *g*_Al-O_ became broader, while those turned narrower in *g*_O-O_, which could be observed more intuitively in Table 2. Simultaneously, for the configurations at zero pressure or with a constant density (*ρ* = 2.81 g/cm^3^ or *ρ* = 3.17 g/cm^3^), changes in *r_αβ_* also happened as the temperature increased, with *r*_Al-Al_ increasing and *r*_Al-O_ decreasing. Regarding *r*_O-O_, it increased along with the temperature for the zero-pressure densities, whereas it was found to be declined when at a constant density. Additionally, for all the Al-O, Al-Al, and O-O correlations, the FWHM of the initial peaks grew along with the temperature. Compared to those at 3000 K, the intensities of the initial peaks of all the *g_αβ_*(*r*) were shown to be lower at 4000 K.

Table 3 demonstrated that for the atom pairs of Al-O, the average coordination numbers (*Z_αβ_*) were approximately located at ~4, consistent with the experimental, ab initio and MD simulation findings. For all the atom pairs of *α-β*, *Z_αβ_* increased along with the density at each temperature. However, when at zero pressure or a fixed density, *Z_αβ_* roughly decreased as the temperatures rose from 2500 K to 4000 K.

**Figure 3:**
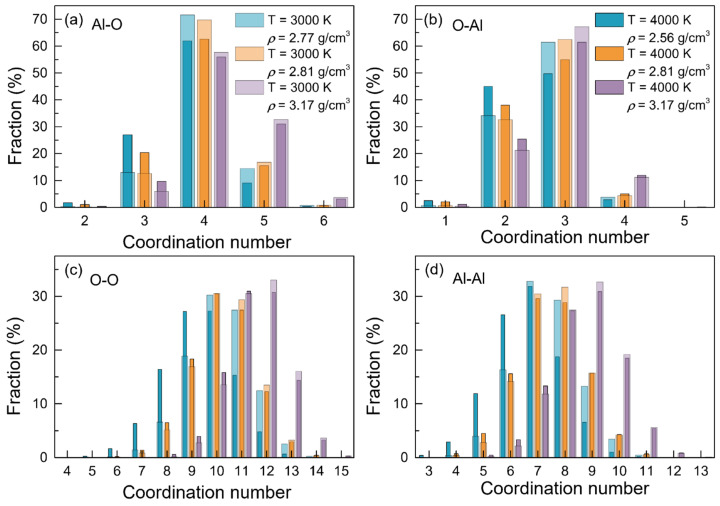
Coordination number distributions for the configurations of liquid alumina with three densities respectively at 3000 K and 4000 K.

Figure 3 displays the coordination number distributions for the atom pairs of Al-O, O-Al, O-O and Al-Al, respectively, at 3000 K and 4000 K. It can be observed that for the Al-O pairs, the coordination numbers (Figure 3a) were distributed between 2 and 6. Besides AlO_4_, there was also a certain amount of AlO_3_ and AlO_5_ present. With the temperature increasing, the fractions of AlO_2_ and AlO_3_ increased, whereas those of AlO_4_, AlO_5_ and AlO_6_ decreased, demonstrating a structural transition on AlO_n_. Moreover, when at a fixed temperature, some changes in the coordination number on AlO_n_ also can be observed as the density rose, with a general decrease in the fractions of AlO_3_ and AlO_4_, accompanying an increase in those of AlO_5_, indicating the trend of structural transition on the elemental units from a tetrahedral network to an octahedral network. These variations in the percentages of AlO_n_ enabled the *Z*_Al-O_ to decrease with increasing temperature, and to increase along with density, in accordance with the findings in Table 3. The coordination numbers of O-Al (Figure 3b) also had a narrow distribution, with a range between 1 and 5. In contrast, the coordination numbers for O-O (Figure 3c) and Al-Al (Figure 3d) exhibited broader ranges, with 4 to 15, and 3 to 13, respectively.

**Figure 4:**
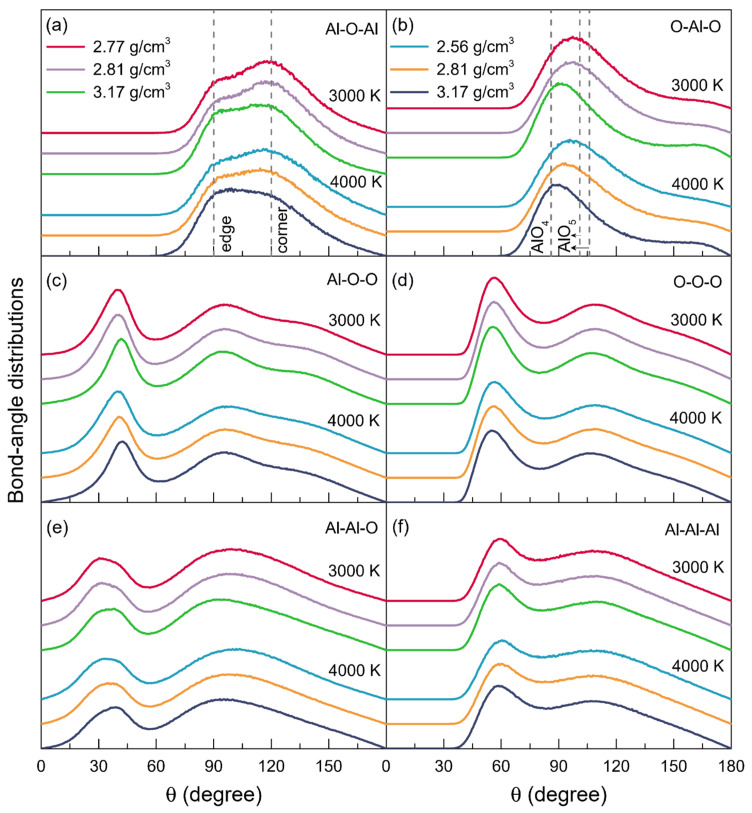
Bond-angle distributions for the models of liquid alumina with three densities at 3000 K and 4000 K, respectively.

The bond-angle distributions, as seen in Figure 4, revealed details about the polyhedral geometry and packing within the structure of liquid alumina. The bond-angel distributions of O-Al-O and Al-O-Al, respectively, can be applied to examine the intra- and inter-polyhedral connectivity. According to Skinner’s experiment [29], the presence of AlO_4_ was associated with peaks around 101° and 106° in the bond-angle distribution of O-Al-O, while AlO_5_ corresponded to the main peak at 86°, following a minor one over the degrees of 140–170°. As shown in Figure 4b, at 3000 K, the main peaks in the bond-angle distributions of O-Al-O, located around 97.17°, 97.02°, and 91.75°, respectively, for *ρ* = 2.77 g/cm^3^, *ρ* = 2.81 g/cm^3^, and *ρ* = 3.17 g/cm^3^. With the temperature increasing to 4000 K, these peaks moved to the smaller degrees of 95.80° for *ρ* = 2.56 g/cm^3^, 93.44° for *ρ* = 2.81 g/cm^3^, and 89.25° for *ρ* = 3.17 g/cm^3^. These O-Al-O angles, together with the *Z*_Al-O_ that was equal to about 4, characterized the liquid alumina as being composed of disordered (AlO_4_)^5-^ tetrahedral units. Moreover, it could be inferred that the initial peaks in the bond-angle distributions of O-Al-O, which ranged from 89.25° to 97.17°, should be primarily attributed to the existence of both AlO_4_ and AlO_5_. While at 3000 K or 4000 K, with the density increasing, the initial peaks shifted toward smaller degrees, and the minor peaks at about 165° intensified, both of which indicated the enhancing proportions of AlO_5_, in line with the findings of the Al-O coordination number distributions (Figure 3a). Meanwhile, as the temperature increased, the initial peaks also changed to lower degrees, which might be due to the relatively high AlO_3_ percentages.

The Al-O-Al bond-angle distributions at 3000 K and 4000 K are shown in Figure 4a. At both 3000 K and 4000 K, there were two main peaks in each Al-O-Al bond-angle distribution: the one approximately distributed at 93° corresponded to the linkages of AlO_5_-AlO_5_ or AlO_5_-AlO_4_ by edge-sharing [29], while the other one located at 119° was attributed to the oxygen atoms that were three-fold coordinated, and linked by their corners to AlO_4_ or AlO_5_ [29]. The intensity ratio between the peak around 93° and the one at 119° (*I*_93°_/*I*_119°_) increased along with the density or temperature, indicating the increasing fractions of the edge-sharing networks.

### 3.2. Vibrational Properties

**Figure 5:**
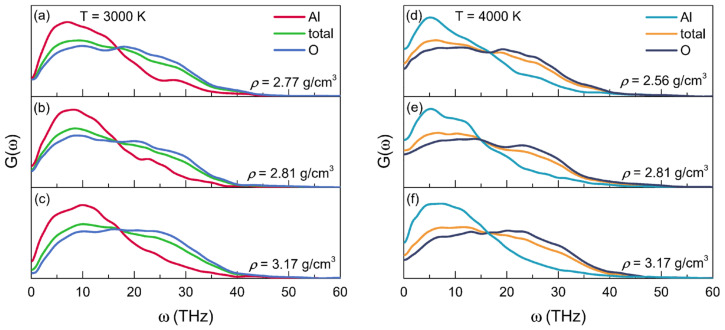
Partial and total vibrational density of states (VDOS) for the configurations of liquid alumina at (**a**–**c**) 3000 K and (**d**–**f**) 4000 K, with three densities at each temperature.

Figure 5 displays the partial and total VDOSs for the configurations of liquid alumina with three densities, respectively, at 3000 K and 4000 K. It is clear that the VDOS spectra at 3000 K ranged from 0 to 50 THz, while those at 4000 K had wider ranges, up to about 55 THz. There were two principal bands in each VDOS spectrum, divided at frequencies between 15 and 20 THz. In accordance with the findings of Vashishta et al. [35], the Al atom vibrations largely produced the band with lower frequencies, whereas the O atom vibrations provided the band with higher frequencies. Additionally, similar to those for other amorphous networks that were predominantly made up of basic tetrahedrons [63], both the modes of bond-bending and vibrations of inter-tetrahedra were related to the lower frequency bands, while the bonding-stretching modes and vibrations of intra-tetrahedra were associated with the higher frequency bands [64].

### 3.3. Transport Properties

#### 3.3.1. Self-Diffusion Coefficient

**Figure 6:**
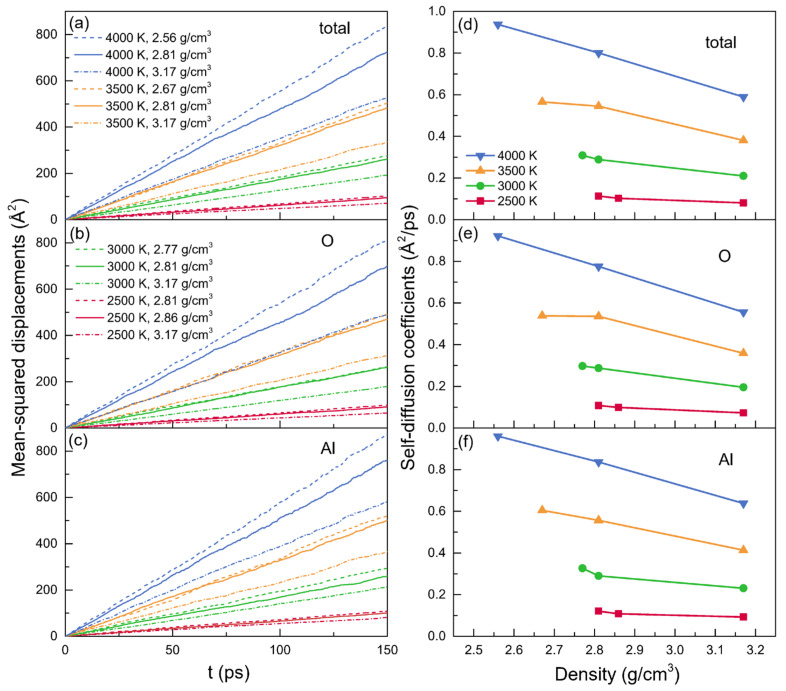
(**a**–**c**) The mean-squared displacements (MSDs) and (**d**–**f**) the averaged self-diffusion coefficients (*D*) for liquid alumina at temperatures of 2500 K, 3000 K, 3500 K and 4000 K, with three densities at each temperature.

The time dependence of MSDs and the density dependence of self-diffusion coefficients at temperatures of 2500 K, 3000 K, 3500 K, and 4000 K are illustrated in Figure 6. Al atoms moved more quickly than O atoms when they were present at the same density and temperature, as evidenced by the self-diffusion coefficients of Al atoms being slightly larger than that of O atoms, as shown in Table 4. The distinct diffusion mechanisms that occurred between Al and O particles could be able to explain this phenomenon. In addition to being in the free states, the breakdown or reformation of Al-O bonds influenced the diffusions of Al particles, while the transitions between the bridging and the nonbridging, often needing higher energies, affected the diffusions of O particles [65]. Table 4 displays the numerical values for the self-diffusion coefficients. Significantly, the results for the configuration at 2500 K and 2.81 g/cm^3^ agreed well with those calculated by Jahn et al. [66] using MD simulations based on an advanced ionic model type potential. For the models at zero pressure or with a fixed density, the self-diffusion coefficients increased along with the temperature. Meanwhile, it also can be observed that the self-diffusion coefficients generally decreased with density increasing.

#### 3.3.2. Thermal Conductivity

**Figure 7:**
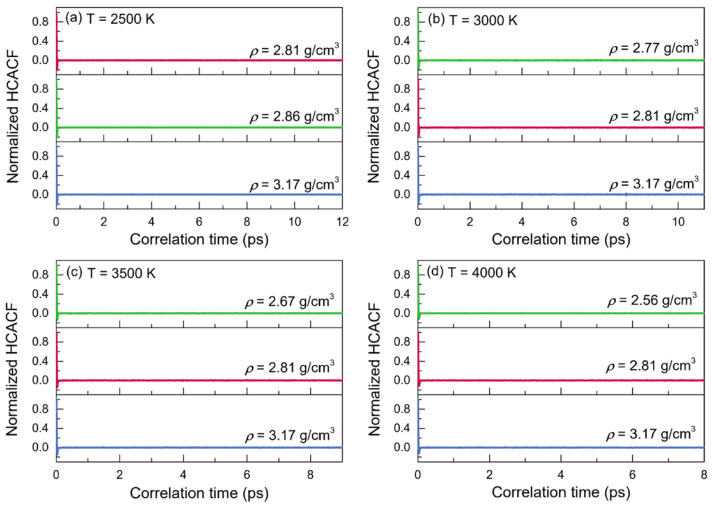
Normalized heat-current autocorrelations (HCACFs) for the configurations of liquid alumina with three different densities at (**a**) 2500 K, (**b**) 3000 K, (**c**) 3500 K and (**d**) 4000 K, respectively.

**Figure 8:**
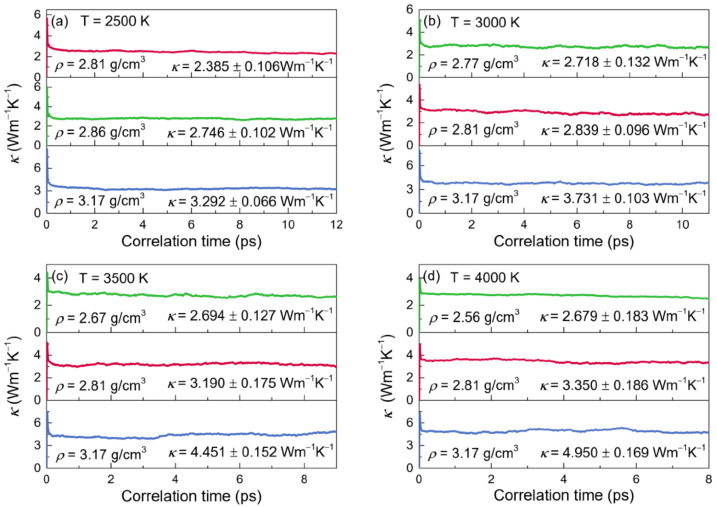
Thermal conductivities (*κ*) for the configurations of liquid alumina with three different densities at (**a**) 2500 K, (**b**) 3000 K, (**c**) 3500 K and (**d**) 4000 K, respectively.

The normalized heat-current autocorrelation functions (HCACFs) and thermal conductivities (*κ*) of liquid alumina are given as functions of correlation time in Figure 7 and Figure 8, respectively, at temperatures of 2500 K, 3000 K, 3500 K and 4000 K. Arima et al. reported that the thermal conductivities would be better estimated when the corresponding normalized HCACFs settled to zero or a constant value [67]. The normalized HCACFs in this investigation gradually decreased to negative until ultimately decaying to zero (Figure 7), confirming the reliability of the simulated thermal conductivity results.

It took a few picoseconds, as shown in Figure 8, for the curves of *κ* to stabilize at a specified value, which was accepted to be the value of thermal conductivity. As the influence of phonons grew with the temperature, the curves of *κ* required less time to reach the constant values, and the correlations dissipated more quickly as well. These findings matched those reported by Alexander et al. [68], who investigated the thermal conductivities for the actinide dioxides of UO_2_, and ThO_2_ using the EMD calculations. Thus, by averaging the related curves from 6 to 12 ps at 2500 K, from 3 to 11 ps at 3000 K, from 3 to 9 ps at 3500 K and from 3 to 8 ps at 4000 K, the thermal conductivity values were determined. When at a fixed temperature, *κ* enhanced with density increasing. Meanwhile, it raised along with temperature while at the density of 2.81g/cm^3^ or 3.17 g/cm^3^, but declined as the temperature increased for the zero-pressure densities.

#### 3.3.3. Viscosity

**Figure 9:**
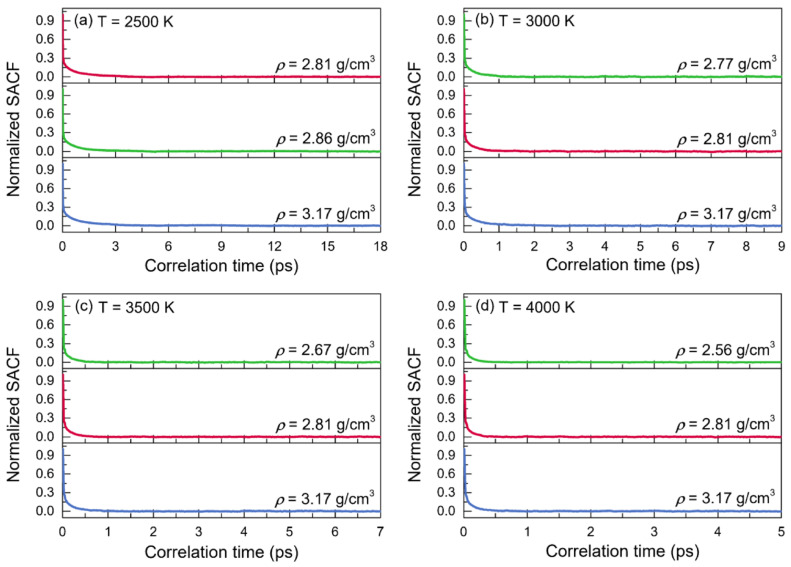
Normalized stress autocorrelation functions (SACFs) for the configurations of liquid alumina with three different densities at (**a**) 2500 K, (**b**) 3000 K, (**c**) 3500 K, and (**d**) 4000 K, respectively.

**Figure 10:**
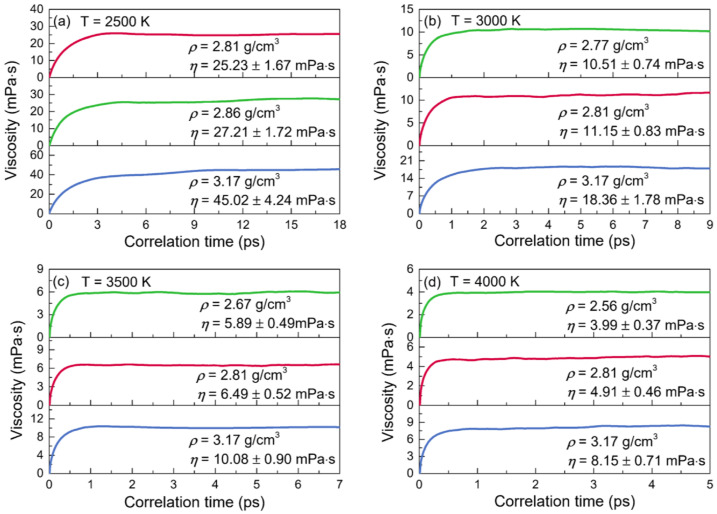
Viscosities (*η*) for the configurations of liquid alumina with three different densities at (**a**) 2500 K, (**b**) 3000 K, (**c**) 3500 K, and (**d**) 4000 K, respectively.

The normalized stress autocorrelation functions (SACFs) and viscosities (*η*) for the configurations of liquid alumina at temperatures from 2500 K to 4000 K are shown in Figure 9 and Figure 10, respectively. The normalized SACF curves fell monotonically and quickly declined to zero, taking just 6 ps at 2500 K, 2.5 ps at 3000 K, 1.5 ps at 3500 K, and even less than 1 ps at 4000 K. Similar to the cases of the thermal conductivities, the time taken by the viscosities curves to reach a stable value decreased with the temperature increasing, along with the decline of the duration at this stable value. Therefore, the viscosity values of *η* were achieved by averaging the values between 11 and 18 ps at 2500 K, between 3 and 9 ps at 3000 K, between 2 and 7 ps at 3500 K, and between 1 and 5 ps at 4000 K. As can be seen, the viscosity values increased along with density at each temperature. Moreover, for the configurations at zero pressure or with a constant density, they declined quickly when the temperature increased. Moreover, it was worth noting that the predicted viscosity value of 25.23 mPa·s at 2500 K with the density of 2.81g/cm^3^, was in good accordance with the experimental value of 25.6 mPa·s given by Paradis et al. [32], who tested the viscosities of molten alumina by the electrostatic levitation and multi-beam radiative heating techniques. Furthermore, the calculated viscosities of the models at 2500 K with densities of 2.81 g/cm^3^ and 2.86 g/cm^3^, as well as those at 3000 K with densities of 2.77 g/cm^3^ and 2.81 g/cm^3^ (Figure 10), were all very close to the results obtained by the density-function theory based on electronic structure calculations [69].

## 4. Conclusions

Using molecular dynamics simulations, the structural, vibrational, and transport characteristics of liquid alumina were investigated, with the temperature increasing from 2500 K to 4000 K. Configurations with densities of 2.81 g/cm^3^ and 3.17 g/cm^3^, as well as the relaxed ones with nearly zero pressure, were explored at intervals of 500 K. Transformations in structural, vibrational, and transport characteristics were discussed in relation to the changes in temperatures or densities.

The newly optimized parameters associated with the Born–Mayer–Huggins potential with were taken to calculate the atomic interactions. Although they were originally developed for aluminosilicate (AS), the findings of this paper proved their efficiency in characterizing the structure and some dynamical features of the pure liquid alumina.

The structure of liquid alumina was primarily made up of the slightly disordered AlO_4_ tetrahedral units, with tiny fractions of AlO_2_ and AlO_6_, as well as a certain amount of AlO_3_ and AlO_5_. Structural transitions on the elemental units occurred as either the temperature or density increased. The explorations of the intermediate-range order revealed the two primary approaches that the elemental units being connected to each other: sharing edge or corner.

By applying the Fourier transform to the velocity autocorrelation function, the vibrational density of states (VDOS) was calculated, which presented two principal frequency bands, one with the higher frequency bands from the vibrations of O atoms, relating to the bonding-stretching modes and vibrations of intra-tetrahedra, while the other with the lower frequency ones from the vibrations of Al atoms, associating with the modes of bond-bending and vibrations of inter-tetrahedra.

The self-diffusion coefficient (*D*) was estimated by the linear behavior of the mean-squared displacement over a long time, and by using the Green–Kubo relation, the thermal conductivity (*κ*) and viscosity (*η*) were calculated through the EMD simulations. When at a fixed temperature, the thermal conductivities and viscosities increased with the density, although the self-diffusion coefficients for both Al and O particles decreased. Meanwhile, changes in temperatures also affected the transport coefficients. For the models with the density of 2.81g/cm^3^ or 3.17 g/cm^3^, with the increasing temperature, *D* and *κ* were discovered to be enhanced, whereas *η* declined, while for the zero-pressure models, *D* also increased along with the temperature, but *κ* and *η* decreased. Notably, the viscosity at 2500 K with the density of 2.81 g/cm^3^ equaling 25.23 mPa·s was in line with the experimental finding of 25.6 mPa·s.

## Figures and Tables

**Table 1:** Potential parameters for liquid alumina [48].

	*q_α_*_(*β*)_	*A_αβ_* (eV)	*ρ_α_*_β_ (Å)	*σ_αβ_* (Å)	*C_αβ_* (eV·Å6)
Al	1.8				
O	−1.2				
Al-Al		0.0029	0.0680	1.5704	14.0498
Al-O		0.0075	0.1640	2.6067	34.5747
O-O		0.0120	0.2630	3.6430	85.0840

**Table 2:** Position (*r_αβ_*) and full width at half maximum (FWHM) of the initial peak in partial pair distribution functions for liquid alumina from this MD simulation and the data from experiments and ab initio simulation.

Density (g/cm^3^)	Temperature (K)	*r_αβ_* (Å)			FWHM (Å)		
		Al-Al	Al-O	O-O	Al-Al	Al-O	O-O
zero-pressure	2500	3.1723	1.7657	2.7849	0.571	0.219	0.499
	3000	3.1977	1.7618	2.7993	0.610	0.234	0.520
	3500	3.2125	1.7590	2.8021	0.640	0.247	0.545
	4000	3.2303	1.7551	2.8127	0.671	0.259	0.569
2.81	2500	3.1853	1.7648	2.8006	0.566	0.218	0.491
	3000	3.1944	1.7632	2.7922	0.612	0.235	0.513
	3500	3.2100	1.7614	2.7837	0.654	0.247	0.532
	4000	3.2066	1.7589	2.7764	0.684	0.260	0.553
3.17	2500	3.1551	1.7721	2.7368	0.607	0.224	0.460
	3000	3.1611	1.7705	2.7257	0.648	0.238	0.477
	3500	3.1743	1.7670	2.7182	0.674	0.249	0.491
	4000	3.1644	1.7653	2.7087	0.701	0.262	0.505
Data from experiments, ab initio and MD simulations							
experiments	2587 [61]	3.09	1.80	2.76			
	2500 [24]	3.25	1.78 ± 0.05	2.84			
	2400 [29]	3.15	1.80	2.82			
ab initio and MD simulations	3000 [41]	3.14	1.70	2.78			
	2500 [40]	3.20	1.77	2.80			
	2350 [62]	3.14	1.73	2.80			

**Table 3:** The average coordination number (*Z_αβ_*) for liquid alumina from this MD simulation and the data from experiments and ab initio simulation.

Density (g/cm^3^)	Temperature (K)	*Z_αβ_*			
		Al-Al	Al-O	O-Al	O-O
zero-pressure	2500	7.83	4.15	2.76	10.71
	3000	7.42	4.02	2.68	10.24
	3500	7.12	3.91	2.61	9.85
	4000	6.75	3.79	2.53	9.40
2.81	2500	7.59	4.10	2.73	10.48
	3000	7.57	4.06	2.70	10.37
	3500	7.53	3.99	2.66	10.31
	4000	7.49	3.94	2.63	10.26
3.17	2500	8.78	4.36	2.91	11.61
	3000	8.75	4.34	2.89	11.57
	3500	8.66	4.29	2.86	11.46
	4000	8.64	4.27	2.85	11.45
Data from experiments, ab initio and MD simulations					
experiments	2700 [61]		4.37		
	2500 [24]		4.2 ± 0.3		
	2416 [61]		4.39		
	2400 [29]	8.85	4.40	2.93	12.90
ab initio and MD simulations	3000 [41]	7.93	4.31	2.87	10.54
	2500 [40]	8.00	4.20	2.80	7.44
	2350 [62]		4.5		

**Table 4:** Self-diffusion coefficients for liquid alumina at the temperature ranging from 2500 K to 4000 K.

Density (g/cm^3^)	Temperature (K)	Self-Diffusion Coefficients (Å^2^/ps)		
		Al	O	Total
zero-pressure	2500	0.1083 ± 0.0023	0.0989 ± 0.0039	0.1027 ± 0.0031
	3000	0.3264 ± 0.0019	0.2972 ± 0.0037	0.3089 ± 0.0029
	3500	0.6047 ± 0.0028	0.5386 ± 0.0035	0.5652 ± 0.0032
	4000	0.9610 ± 0.0036	0.9219 ± 0.0026	0.9376 ± 0.0029
2.81	2500	0.1205 ± 0.0017	0.1079 ± 0.0029	0.1131 ± 0.0022
	3000	0.2898 ± 0.0038	0.2875 ± 0.0021	0.2886 ± 0.0025
	3500	0.5568 ± 0.0027	0.5359 ± 0.0024	0.5448 ± 0.0024
	4000	0.8367 ± 0.0042	0.7756 ± 0.0031	0.8010 ± 0.0032
3.17	2500	0.0928 ±0.0038	0.0728 ± 0.0026	0.0809 ± 0.0028
	3000	0.2309 ± 0.0046	0.1956 ± 0.0039	0.2099 ± 0.0041
	3500	0.4135 ±0.0029	0.3586 ± 0.0036	0.3806 ± 0.0031
	4000	0.6379 ± 0.0032	0.5557 ± 0.0025	0.5891 ± 0.0026

## Data Availability

The parameters for the Born–Mayer–Huggins potential used in this manuscript were reported in Bouhadja’s paper, defined in the references section as [48]. New data are available from the corresponding authors upon reasonable request.
